# Integrated mRNA and miRNA transcriptome reveal a cross-talk between developing response and hormone signaling for the seed kernels of Siberian apricot

**DOI:** 10.1038/srep35675

**Published:** 2016-10-20

**Authors:** Jun Niu, Jia Wang, Jiyong An, Lili Liu, Zixin Lin, Rui Wang, Libing Wang, Chao Ma, Lingling Shi, Shanzhi Lin

**Affiliations:** 1 College of Biological Sciences and Biotechnology, National Engineering Laboratory for Tree Breeding, Key Laboratory of Genetics and Breeding in Forest Trees and Ornamental Plants, Ministry of Education, Beijing Forestry University, Beijing 10083, China; 2 Research Institute of Forestry, Chinese Academy of Forestry, Beijing 10091, China

## Abstract

Recently, our transcriptomic analysis has identified some functional genes responsible for oil biosynthesis in developing SASK, yet miRNA-mediated regulation for SASK development and oil accumulation is poorly understood. Here, 3 representative periods of 10, 30 and 60 DAF were selected for sRNA sequencing based on the dynamic patterns of growth tendency and oil content of developing SASK. By miRNA transcriptomic analysis, we characterized 296 known and 44 novel miRNAs in developing SASK, among which 36 known and 6 novel miRNAs respond specifically to developing SASK. Importantly, we performed an integrated analysis of mRNA and miRNA transcriptome as well as qRT-PCR detection to identify some key miRNAs and their targets (miR156-SPL, miR160-ARF18, miR164-NAC1, miR171h-SCL6, miR172-AP2, miR395-AUX22B, miR530-P2C37, miR393h-TIR1/AFB2 and psi-miRn5-SnRK2A) potentially involved in developing response and hormone signaling of SASK. Our results provide new insights into the important regulatory function of cross-talk between development response and hormone signaling for SASK oil accumulation.

In recent years, the seed oils of woody plants with a notable advantage over conventional feedstocks have been used as potential raw materials for biodiesel production in China^1^. Thus, it is important to develop non-food plant resources for biodiesel in China.

Siberian apricot (*Prunus sibirica* L.), belonging to family Rosaceae and the genus *Prunus*, is native to the temperate and mountainous districts of northern and northeastern China, eastern and southeastern regions of Mongolia, eastern Siberia regions and maritime territory of Russia^2^. In China, Siberian apricot is one of the most economically and ecologically important and intensively studied woody oil plants owing to its very plentiful resource, superior adaptability and ecological benefits^3^. Based on our previous evaluations of biodiesel yield, oil content, fatty acid (FA) composition, cold filter plugging point, cetane number, oxidative stability and flash point of Siberian apricot seed kernels (SASK) oil, Siberian apricot has been characterized as a novel feedstock for biodiesel production in China^1^,^4^,^5^,^6^. Recently, several studies have shown an involvement of miRNAs in posttranscriptional regulation for seed or fruit development of soybean, peanut, rapeseed, strawberry, *Solanum lycopersicum*, *Citrus sinensis*, *Vitis vinifera*, *Lagenaria siceraria*, *Ricinus communis*, *Carthamus tinctorius* and *Jatropha curcas*^7^,^8^,^9^,^10^,^11^,^12^.

In plants, microRNAs (miRNAs), endogenous single-stranded small non-coding RNAs, have been shown to serve as negative regulators to modulate gene expression at post-transcriptional level by transcript cleavage or translational repression of target genes, and play essential roles in the development of plants^13^. In recent years, RNA sequencing, as one next-generation sequencing technology, has become an effective choice for miRNA transcriptome study in some oilseed plants^14^,^15^,^16^,^17^,^18^. However, up to now reports about this situation for woody plants only in *J. curcas*, olive and oil palm^19^,^20^,^21^. Thus, miRNA transcriptomic analysis of developing SASK has become an imperative for the development of woody biodiesel.

Recently, our transcriptomic analysis has identified some functional genes responsible for oil biosynthesis in developing SASK^22^. However, the complex molecular regulatory mechanism for SASK oil accumulation was still poorly understood. In this continued study, the dynamic patterns of growth tendency (weight and size) and oil content of SASK were detected at 7 different developing stages (10, 20, 30 40, 50, 60, and 70 days after flowering (DAF)), and then the 3 representative SASK samples at 10 DAF (immature), 30 DAF (middle) and 60 DAF (mature) were selected for small RNA (sRNA) sequencing, respectively. The resulting unique sRNA sequences from 3 sRNA libraries of developing SASK were compared with miRBase and peach genome, and all our identified known and novel SASK miRNAs were used for functional predication and differential expression analysis. Moreover, some key miRNAs and their targets, involved specifically in hormone signaling pathways and the SASK growth, development and oil accumulation, were characterized by using a combination of mRNA and miRNA transcriptomic study as well as qRT-PCR analysis. All our findings provide new insights into the important regulatory function of cross-talk between development response and hormone signaling for SASK oil accumulation, which will contribute to reveal the complex network regulatory mechanism of SASK oil accumulation at transcriptional and posttranscriptional levels for the development of woody biodiesel.

## Results

### SASK growth and oil accumulation involved specifically in different developing response signals

**Figure 1:**
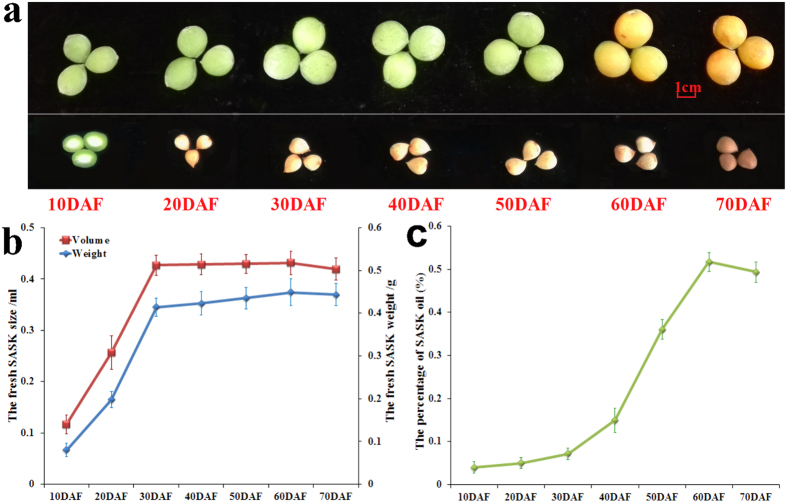
The developmental stages of Siberian apricot. (**a**) The fruits and kernels of Siberian apricot from 7 developing stages. The kernels at 10 DAF could not be segregated wholly from the fruits. (**b**) The growth tendency of kernels at 7 different development periods. Error bars indicate standard deviations of five biological replicates. (**c**) The oil content of SASK at 7 different development periods.

To explore whether or not the growth and oil accumulation of SASK specifically responded to different developing signals, we systematically measured the dynamic patterns of growth tendency (weight and size) and oil content of SASK during the whole developmental stages from 10 DAF (immature stage) to 70 DAF (fully matured stage). The weight of SASK was approximately 4.1-fold higher at 30 DAF (0.41 ± 0.02 g) than at 10 DAF (0.08 ± 0.01 g), and 7.14% increase was observed from 40 DAF (0.42 ± 0.03 g) to 60 DAF (0.45 ± 0.03 g), followed by 4.44% decline at 70 DAF (0.43 ± 0.04 g), which was consistent with the dynamic changes of SASK size in developing stages (Fig. 1ab), showing indeed that the growth and development of SASK was mainly at the early-middle stage (10–30 DAF). However, we observed that the oil content of SASK exhibited a rapid accumulation (approximate seven fold) from 7.16 ± 1.38% at 30 DAF to 51.68 ± 2.18% at 60 DAF (Fig. 1c), which was in accordance with our previous report^22^, indicating a higher oil accumulation for developing SASK at middle-late stage (30–60 DAF). Thus, our investigations revealed that the growth and oil accumulation of developing SASK may specifically respond to different developmental signals.

To better explore the posttranscriptional regulatory mechanism of miRNAs involved in SASK development and oil accumulation, 3 SASK samples (10, 30 and 60 DAF) at critical periods of growth and oil accumulation (Fig. 1b,c) were selected as the experimental samples for comparative deep sRNA transcriptomic analysis.

### Identification of known and novel miRNAs in developing SASK by sRNA sequencing

**Figure 2:**
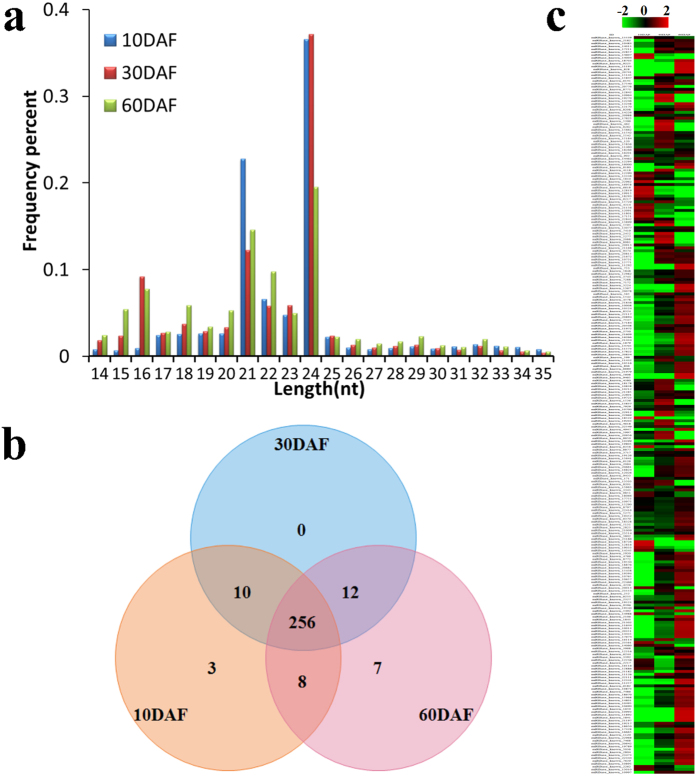
Identification of SASK miRNAs in developing SASK by sRNA sequencing. (**a**) Length distributions of sRNA sequences identified in 3 the sRNA libraries. (**b**) The distribution of known miRNAs in 3 developing stages. (**c**) The heatmaps of SASK miRNAs at 3 developmental stages. The detailed sequences were showed in Tables S4.

To explore the miRNAs-mediated regulation in developing SASK, 3 separate sRNA libraries from 3 developing stages (10, 30 and 60 DAF) were analyzed by Illumina Hiseq2500, and 7,336,170 (1,905,120 unique reads), 7,284,101 (2,116,046) and 7,437,072 (1,599,564) clean reads were respectively generated (Table 1). Subsequently, we performed the quality evaluation of our sRNA sequencing data by the FastQC software, and identified those generated clean reads with high quality for the further analysis of SASK miRNAs (Additional File 1: Fig. S1). Notably, the majority of sequences (70.70% at 10 DAF, 61.07% at 30 DAF and 48.84% at 60 DAF) was identified within 21–24 nt (Fig. 2a), which was consistent with the typical length distribution pattern of sRNAs for *Prunus mume*^23^ and *Prunus persica*^24^. Moreover, 1,769,760 (24.12%), 1,822,295 (25.02%) and 2,811,012 (37.80%) clean reads obtained respectively from 3 sRNA libraries (10, 30 and 60 DAF) were annotated function, among which 974,329 (13.28%), 478,734 (6.57%) and 564,848 (7.60%) reads were assigned to miRNA (Additional File 2: Table S1). By categorized analysis, we obtained 296 known miRNAs, of which 256 miRNAs were shared in 3 developmental stages, but 40 miRNAs were identified to be development-specific (Fig. 2b,c). Additionally, by miRNA family clustering analysis, all known SASK miRNAs were belonged to 88 miRNA families (Additional File 3: Table S2), among which 20 miRNA families (miR156, miR159, miR160, miR162, miR164, etc.) were highly conserved with Arabidopsis, *Oryza sativa*, and *Populus trichocarpa*^13^, indicating that these conserved miRNAs may play the fundamental regulations in developing SASK. However, the other known miRNA families (miR858, miR7122, miR6285, miR6284, miR6281, miR482, etc.) were characterized to be conserved among *Prunus* species (the same family as our experimental material), suggesting that they may be *Prunus*-specific miRNAs.

As surprised by 5,566,410 (75.88%), 5,461,806 (74.98%) and 4,626,060 (62.20%) non-annotated reads respectively from 10, 30 and 60 DAF of SASK (Additional File 2: Table S1), we attempted to establish whether or not novel miRNAs was involved in development and oil accumulation of SASK. To achieve this, all the non-annotated reads from the 3 SASK sRNA libraries were aligned with *P. persica* genome sequence, and then miRDeep and RNAfold software were used to predict potential novel miRNAs and their secondary structures, respectively^25^. The resulting 44 potential novel pre-miRNAs (designed as psi-miRn1 to psi-miRn44) was identified with minimal folding energy (−30.05 kcal/mol) in developing SASK (Additional File 4: Table S3 and Additional File 5: Fig. S2), indicating that all the precursors of our predicted novel SASK miRNAs could be folded into stable hairpin-like structures. Notably, 22 novel SASK miRNAs (psi-miRn1 to psi-miRn22) were identified with complementary miRNA* sequences, and most of them were found with far less reads than mature miRNAs (Table 2), probably owing to the degradation of miRNA*s in biosynthesis of pathway miRNA^13^. Thus, the existence of miRNA*s prompt us to believe the authenticity of predicted novel miRNAs.

To our knowledge, this is the first study of miRNA transcriptomic analysis in developing SASK, and our characterized known and novel miRNAs will provide the important foundation to further deeply explore miRNA-mediated regulatory mechanism for SASK development and oil accumulation.

### A broad range of biological functions for the targets of known and novel miRNAs in developing SASK

Recently, we have completed mRNA transcriptomic analysis of developing SASK^22^, which could be as the important references for functional predictions of SASK miRNAs by identifying their targets. Here, 281 known miRNAs and 41 novel miRNAs as well as 8 miRNA*s from developing SASK were predicted to target 2240 genes by using psRNATarget (Additional File 6: Table S4). All these identified targets were analyzed by using BLASTX against protein databases of Swissprot, Cdd, Tremble, NR and PFAM, followed by GO and KEGG analysis. The resulting 65.19% (1460) of all predicted targets (2240) for SASK miRNAs showed significant similarity to the known proteins in at least one database (Additional File 6: Table S4).

Generally, miRNAs play the important roles by posttranscriptional regulation of their target genes, and many transcription factors (TFs) as the targets of miRNAs were identified to be related to development and oil biosynthesis of plant seeds, such as miR156 targeted for squamosa promoter binding protein-like (SPL), miR159 for MYB transcription factor, miR164 for NAC domain-containing protein, miR160 for auxin response factor (ARF), miR171 for scarecrow-like protein 6 and miR172 for APETALA2 (AP2)^13^,^26^,^27^. Indeed, we characterized SASK miRNAs to target various groups of TFs, including homeobox-leucine zipper protein ATHB by miR166, SPL by miR156, MYB by miR159, ARF by miR160, NAC1 by miR164, nuclear transcription factor Y by miR169, AP2 by miR172, WRKY7 by psi-miRn44, WIN1 by psi-miRn24 and MYB by psi-miRn31 (Additional File 6: Table S4), suggesting the master regulation of miRNA-targeted TFs in developing SASK. Impressively, many predicted non-TF targets of some miRNAs were also identified in developing SASK (Additional File 6: Table S4), such as sulfate transporter, rhodenase, laccase, ubiquitin conjugating enzyme, peptide/nitrate transporter, peptidyl-prolyl cis-trans isomerase, elongation factor 1-delta 2, decapping protein 5 and BAG family molecular chaperone regulator 6 (BAG6) by miR395, miR396, miR397, miR399, psi-miRn26, psi-miRn29, psi-miRn38, psi-miRn13 and psi-miRn37, respectively. Together, these results indicated that the targets of known and novel miRNAs might play roles in a broad range of biological functions in developing SASK.

### Differentially expressed miRNAs specifically in response to developing SASK

**Figure 3:**
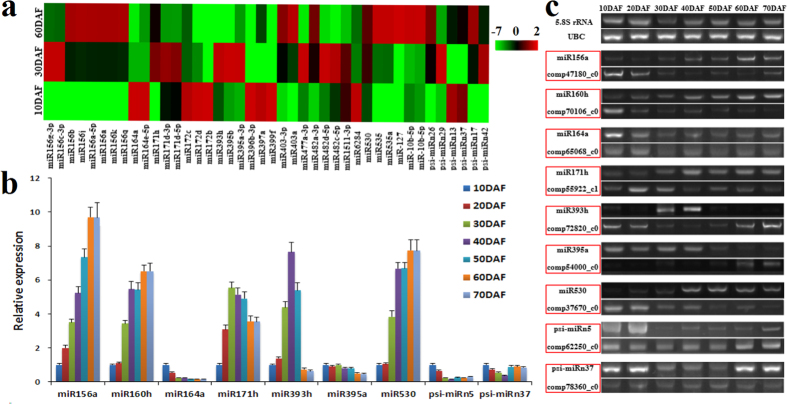
Differentially expressed analysis of miRNAs and their targets. (**a**) The heatmaps of differentially expressed miRNAs in response to development. (**b**) Expression analyses of miRNAs in developing SASK by qRT-PCR. (**c**) Validation of miRNAs and their targets by RT-PCR. The mRNA (top) and its corresponding target (bottom) are shown in each red box. The 5.8S rRNA and *UBC* were used as internal genes respectively for the normalization of miRNAs and their targets.

MiRNA-mediated regulations have been reported to be critical for development or oil accumulation of plant seeds^14^,^15^,^16^,^17^,^18^,^19^,^20^,^21^. To determine the specific SASK miRNAs in response to different developing stages, the differentially expressed miRNAs were compared between 2 libraries from different developing stages. The resulting 30 (18 up-regulation and 12 down-regulation), 5 (4 up-regulation and 1 down-regulation) and 14 (5 up-regulation and 9 down-regulation) miRNAs were identified specifically for 30/10 DAF, 60/10 DAF and 60/30 DAF, respectively (Additional File 7: Table S5). After removing redundant miRNAs, we obtained 42 miRNAs (36 known and 6 novel miRNAs) that were differentially expressed in response to developing SASK (Fig. 3a and Additional File 8: Table S6). These data, combined with our findings on the rapid growth of SASK at early development and the great biosynthesis of oil at middle-late development (Fig. 1b,c), suggested the potential regulatory roles of differentially expressed miRNAs in development and oil biosynthesis of SASK.

Interestingly, a significant difference of up-regulation among different members of a certain miRNA family was found in developing SASK. As for miR156 family, miR156c-3p and miR156g-3p were up-regulated specifically at middle development of SASK, while the others (miR156a, miR156b, miR156a-5p, miR156j, miR156k and miR156q) were up-regulated at middle-late development (Fig. 3a and Additional File 8: Table S6). Also noteworthy for miR171 and miR482 families, the abundant transcripts of miR171b/d, miR482d/c, and miR171h and miR482a were respectively identified at early-late, middle and middle-late development of SASK, respectively (Fig. 3a and Additional File 8: Table S6). These results indicated that different members from the same miRNA family may be expressed at specific developmental stages of SASK, probably be attributed to a cooperative and redundant regulation activity of miRNAs in developing SASK.

### Transcriptional and posttranscriptional regulations of signaling pathways in developing SASK

Plant hormones, as the main messengers in the signaling pathways, are vital to determine development and oil biosynthesis of plants^28^,^29^. To explore whether hormone signaling pathway was involved specifically in developing SASK, the transcript patterns of genes encoding hormone signal transducers were analyzed according to our previous mRNA transcriptomic data^22^. As a result, a total of 167 genes was identified in 8 hormone signaling pathways in developing SASK, of which 56 genes displayed the differential expressions in developing SASK (Additional File 9: Table S7 and Additional File 10: Fig. S3), indicating a complex relationship between developing process and different hormone response signals in SASK at transcriptional level.

Noticeably, miRNA-based posttranscriptional regulation has shown to be involved in hormone signaling of plants^27^. By our function prediction of miRNAs, a total of 14 signal transducers for 5 signaling pathways was predicted as the targets of 10 miRNAs in developing SASK, including two-component response regulators ARR1 (targeted by psi-miRn4) and ARR11 (miR6281) in cytokines signaling, protein phosphatase 2C 37 (P2C37) (miR530) and serine/threonine-protein kinase SnRK2A (psi-miRn5) in abscisic acid (ABA) signaling, cyclin D3-1 and systemin receptor SR160 (miR6281) in brassinolide signaling, TIF6B (miR169) in jasmonic signaling, and ARF17/18 (miR160), F-box protein TIR1/AFB2 (miR393), auxin-induced protein AUX22B (miR395) and auxin influx carrier LAX2 (psi-miRn41) in auxin signaling (Additional File 11: Table S8). Thus, there exists a complex mechanism of miRNA-mediated regulation of hormone signaling pathways in developing SASK. To explore the contribution of miRNA-directed hormone signaling in developing SASK, the differentially expressed miRNAs related to hormone signaling were analyzed. We found a stable low transcript of miR169, miR6281 and psi-miRn4 in developing SASK (Additional File 11: Table S8), implying that miRNA-mediated hormonal signaling pathways (jasmonic, brassinolide and cytokines) may play a little posttranscriptional regulation in developing SASK. Also, a high expression of miR164, miR393, miR395 and miR530 was identified in developing SASK (Additional File 11: Table S8). All these results indicated that miRNA-mediated posttranscriptional regulations may focus mainly on auxin and ABA signaling pathways, thus requiring further analysis.

### Expression analysis of miRNAs and their targets involved in signaling pathway in developing SASK

**Figure 4:**
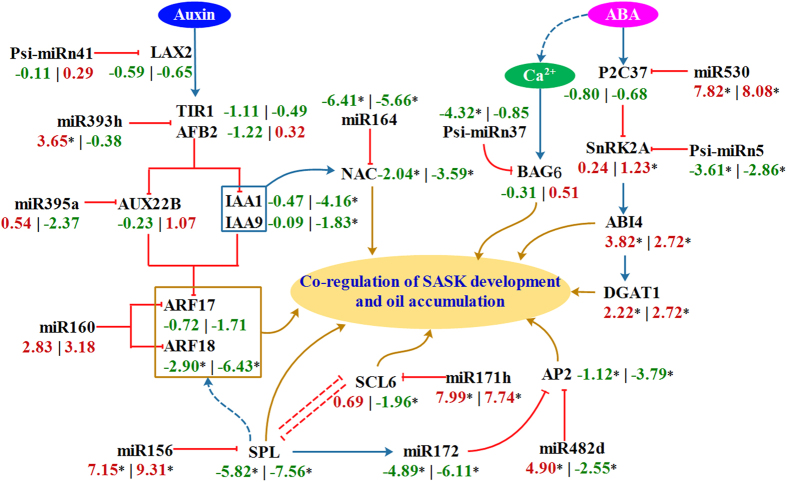
The proposed model of miRNA-mediated regulatory network associated with SASK development and oil accumulation. The blue arrow line means activation, and the red flat line means repression. The values along each enzyme show the relative expressions at 10:30DAF (left) and 10:50DAF (right); red, up-regulation; green, down-regulation. The asterisk (*) indicated the relative expression of the gene with significant difference (*p* value <0.05).

Auxin has an important role in the regulation for the growth and development of plants. To explore the regulatory mechanism of miRNA-mediated auxin signaling pathway in developing SASK, we analyzed the time-course transcript pattern of miRNAs and their targets in this work. We identified auxin influx carrier LAX1 with a high transcript at early development, and LAX2 and 3 with a low expression in developing SASK (Additional File 9: Table S7). However, only LAX2 was predicted as the target of psi-miRn41 (Additional File 11: Table S8), and psi-miRn41 displayed a stable transcript in developing SASK (Table 2). These data suggested that psi-miRn41-meidated regulation could repress the transcript of LAX2, and LAX1 may be as one main auxin influx carrier for SASK at early development. Moreover, the highly expressed TIR1/AFB2 (targeted by miR393h), known as auxin receptor, was observed in early developing SASK, with an inverse transcript pattern of miR393h (Additional File 8: Table S6), which was also supported by our qRT-PCR results from 7 different developing stages of SASK (Fig. 3b,c). These results indicated that the transcript of miR393h-targeted TIR1/AFB2 might be cleaved directly by the miR393h in developing SASK. Additionally, by mRNA transcriptomic analysis, we characterized 7 Aux/IAA proteins (IAA1, 6, 7, 9, and AUX22B, 22D, 28) with the differential expressions in developing SASK, among which IAA1 and 9 were up-regulated specifically at early-middle development (Fig. 4), but the others exhibited a low expression at early development (Additional File 9: Table S7), showing indeed that transcriptional expressions of different Aux/IAA proteins may specifically respond to different developing stages of SASK. It is of interest to note that the up-regulated miR395 (targeted AUX22B) detected in early developing SASK, which was evidenced by our qRT-PCR results from 7 developing stages (Fig. 3b,c), emphasizing that miR395 may be involved in the auxin signaling pathway by the cleavage regulation of AUX22B. Also, 17 ARF members were identified in developing SASK by our mRNA transcriptomic analysis, in which 4 ARF members (ARF2, 17, 18 and 19) and 2 (ARF1 and 9) were up-regulated in early and middle-late development of SASK respectively (Additional File 9: Table S7), suggesting the transcriptional expressions of ARF members in response of SASK to different developing stages. Notably, ARF17/18 was predicted to be targeted by miR160 (Fig. 4 and Additional File 11: Table S8), and thereby the down-regulated miR160 may contribute to transcriptional abundance of ARF17/18 at early development of SASK.

The plant hormone ABA plays a major role in seed growth and development. To investigate the potential role of miRNA-mediated regulation in ABA signaling pathway in developing SASK, the temporal transcript profiles of miRNAs and their targets were comparatively analyzed. Here, from our mRNA transcriptomic analysis, the up-regulated P2C16, 37, 51 and 75 (protein phosphatase 2C family) was identified at early, middle-late and late development, respectively (Additional File 9: Table S7), revealing that the transcripts of different PP2Cs may be involved in the response of SASK to different developmental stages. By our qRT-PCR analysis, we observed the differentially expressed profiles of miR530 (up-regulation) and its target gene (*P2C37*, down-regulation) specifically at middle-late development of SASK (Fig. 3b,c), implying that P2C37 transcript may be involved in miR530-mediated regulation in developing SASK. In addition, 4 members of serine/threonine-protein kinases (SnRK2A, SnRK2C, SnRK2E and SnRK2I) were identified in developing SASK, but only SnRK2A and SnRK2E was up-regulated specifically at middle-late development (Additional File 9: Table S7). Importantly, by a combination of differential expression analysis (Table 2 and Fig. 4) and qRT-PCR detection (Fig. 3b,c), a low transcript for psi-miRn5 (targeted for *SnRK2A* gene) was identified in middle-late developing SASK. These data suggested that ABA-dependent activation of SnRK2A by psi-miRn5 may be responsible for developing SASK.

Also noteworthy was Ca^2+^ as one second messenger involved in a variety of developmental progresses in plants. In the present study, the abundant transcript of psi-miRn37-targeted BAG6 (as Ca^2+^-binding protein) was found predominately in late developing SASK (Fig. 3b,c), suggesting that Ca^2+^ signaling pathway may be involved in psi-miRn37-directed regulation in developing SASK.

### Transcriptional analysis of miRNAs and their targeted TFs in developing SASK

Many TFs as the targets of miRNAs are implicated in the development or oil accumulation of plants. In this work, we performed the function prediction and expression analysis to characterize the differentially expressed miRNAs in developing SASK, most of which were predicated to target TFs (Additional File 8: Table S6), such as SPL2, 4, 6, 13B and 18 (targeted by miR156), NAC1 (miR164), SCL6 (miR171), AP2 (miR172 and miR482d). These data can be interpreted to suggest that miRNA-directed posttranscriptional regulation might be involved in developing SASK. Intriguingly, miR171h and 8 members of miR156 family (miR156a, miR156a-5p, miR156b, miR156c-3p, miR156g-3p, miR156j, miR156k, and miR156q) were identified with a low transcript at early development of SASK (Fig. 3a,b), but miR171h-targeted SCL6 and miR156-targeted SPLs (SPL2, 4, 6, 13B and 18) all displayed the up-regulated expression (Additional File 12: Fig. S4), showing indeed that the transcriptional expressions of *SPLs* and *SCL6* may be cleaved directly by miR156 and miR171h, respectively. Additionally, our mRNA transcriptomic analysis revealed that the homologies of ATVGT1 (sugar transporter family protein, At3g03090), ATK (K^+^ transporter, At4g22200), PGP4 (P-glycoprotein 4, At2g47000), ABCG18 (ABC transporter family protein, At3g55110) and SULTR1;2 (sulfate transporter 1;2, At1g78000) were up-regulated specifically in early-middle stage of developing SASK (Additional File 12: Fig. S4), implying an involvement of membrane protein transported activity in transduction of energy, sugar and inorganic salts responsible for developing SASK. Notably, by the function prediction of miRNAs, we identified miR482d and 3 members (miR172b, miR172c and miR172d) of miR172 family to target the same gene *AP2* in developing SASK (Fig. 4). Interestingly, miR172b/c/d and miR482d displayed a high and low transcript in early developing SASK, respectively (Fig. 3a). These results indicated that the transcriptional expression of AP2 may be involved in the co-regulation by both miR172b/c/d and miR482d in developing SASK.

## Discussion

Siberian apricot (*Prunus sibirica* L.) has been used as raw material for biodiesel in China^4^,^5^,^6^, and our transcriptomic analysis has identified some functional genes responsible for oil biosynthesis in developing SASK^22^. However, the complex regulatory mechanism of SASK oil accumulation is still poorly understood. To better develop SASK oil as woody biodiesel, it is essential to characterize some key miRNAs and their targets involved specifically in growth and oil accumulation of developing SASK. In this study, we firstly analyzed the dynamic patterns of growth tendency (weight and size) and oil content of developing SASK, and characterized the a rapider growth and a higher oil accumulation for developing SASK were mainly at early-middle (10–30 DAF) and middle-late stage (30–60 DAF), respectively (Fig. 1). To explore whether or not miRNA-mediated posttranscriptional regulation were involved in SASK development and oil accumulation, we selected 3 crucial SASK samples (10, 30 and 60 DAF) for comparative deep miRNA transcriptomic analysis for the first time, and 7,336,170, 7,284,101 and 7,437,072 clean reads were respectively generated from 3 developing stages of SASK (Table 1). After the bioinformatics analysis, a total of 296 known and 44 novel miRNAs were identified in developing SASK (Fig. 2c), of which 36 known and 6 novel miRNAs were characterized with differential expression (Fig. 3a). These data could massively fill and enrich the miRNA dataset of Siberian apricot.

Auxin, a major plant hormone, has profound effects on plant development^30^, and four auxin influx carriers (AUX1 and LAX 1–3) can transport auxin into plant cell^31^. In the present work, of our identifed three auxin influx carriers (LAX 1, 2 and 3), only LAX1 displayed a high transcript at early development of SASK (Additional File 9: Table S7). Integrated with a low transcript for LAX2 (targeted by psi-miRn41) in developing SASK (Table 2 and Additional File 11: Table S8) and the rapid growth of SASK specifically at early development (Fig. 1b), we can infer that LAX1 may be as the preferred auxin influx carrier for developing SASK at early stage, and psi-miRn41-mediated regulation of LAX2 may play an important role in auxin transport of developing SASK. It was reported that TIR1/AFB F-box proteins (as auxin receptor) directly link auxin perception to degrade Aux/IAA proteins, resulting in a repression for transcriptional activators of ARF family in Arabidopsis^32^,^33^. Our findings on an inverse correlation between miR393h (down-regulation) and its targeted TIR1/AFB2 (up-regulation) in early developing SASK (Fig. 3b,c) indeed reflected that miR393h-targeted TIR1/AFB2 as dominant auxin receptor may be a limiting factor for auxin response to developing SASK, which was compatible with a previous study in Arabidopsis^32^. In addition, the differentially expressed patterns among 7 Aux/IAA proteins (IAA1, 6, 7, 9, and AUX22B, 22D, 28) was identified in developing SASK (Additional File 9: Table S7), indicating that auxin could induce different Aux/IAA genes to different kinetic expressions in developing SASK, as was reported in Arabidopsis^34^. Interestingly, we found that only AUX22B was predicted to be targeted by miR395 (Additional File 11: Table S8), and an antiparallel transcript pattern of miR395 (up-regulation) and its target (down-regulation) was identified in early developing SASK (Fig. 3b,c), reflecting a core role of miR395-mediated regulation of AUX22B in auxin signaling pathway for developing SASK. As noted previously, the strongly induction of miR164-targeted NAC1 by AUX/IAAs in Arabidopsis^35^, was confirmed in this study, where a coordinated transcript pattern among TIR1/AFB2, IAA1/9 and NAC1 was observed specifically at early-middle development of SASK. Thus, it was concluded that miR164-targeted NAC1, as downstream of miR393-targeted TIR1/AFB2, could be induced by IAA1 and 9 in developing SASK (Fig. 4), which was compatible with the previous study of *NAC1* over-expression in Arabidopsis^36^. This also allowed us to speculate that the regulatory pathway of miR393-TIR1/AFB2 and miR164-NAC1 may play an essential role in auxin signaling for SASK development. Also noteworthy was the downstream genes of Aux/IAA^33^. Here, 17 ARF members were identified with different transcription profiles in developing SASK (Additional File 9: Table S7), suggesting that the overlapping functions of ARF members may be need for proper development of SASK, as was supported by the fact that single *ARF* mutant could not significantly affect growth and development of Arabidopsis^37^. Strikingly, the up-regulated 3 ARF members (ARF2, 18 and 19) showed a reverse correlation with the down-regulated Aux/IAA proteins (AUX22B, AUX22D, AUX28, IAA6 and IAA7) in early developing SASK (Additional File 9: Table S7), showing indeed that the de-repression of the 3 ARFs by Aux/IAA proteins may occur specifically at early development of SASK. Notably, the identification of the oppositely transcriptional pattern of miR160 (up-regulation) and its target ARF18 (down-regulation) in early developing SASK (Fig. 4 and Additional File 11: Table S8), implied the important role of miR160-mediated ARF18 in response to auxin signaling pathway at early development. Recently, ARFs have been shown to regulate growth and metabolite by the activation or repression of auxin-responsive genes, and the inducible effect of auxin treatment on the expressions of oil biosynthetic genes was reported for early developing *C. vulgaris*^28^,^33^. Indeed, we identified a positive correlation among the high expression of ARF2/19 and miR160-targeted ARF18 (Additional File 9: Table S7), the rapid growth of SASK (Fig. 1b), and the significant change of FA compositions at early development^22^. This finding, integrated with our previous observations on the up-regulated KASI, KASII, SAD6 and FAD2/3 related to FA biosynthesis in early developing SASK^22^, implicated that ARF2/19 and miR160-targeted ARF18 may be as the important transcriptional activators in response to auxin signaling for the regulation of SASK growth and FA biosynthesis at early developing stage. Taken together, psi-miRn41 (targeted LAX2), miR160 (ARF 18), miR164 (NAC1), miR393 (TIR1/AFB2) and miR395 (AUX22B) were involved in a complex post-transcriptional regulatory mechanism in auxin signaling pathway for early development and FA biosynthesis of SASK.

ABA has shown to regulate developmental response in plants, and several P2Cs have been identified as negative regulators for ABA response^38^. In the present work, our investigations that four members (P2C16, 37, 51 and 75) of protein phosphatase 2C family were up-regulated specifically in response to different developing stages of SASK (Additional File 9: Table S7), suggested that the overlapping functions of PP2Cs with differential profiles may be involved in ABA response to developing SASK. Impressively, we performed a combination of mRNA transcriptomic analysis and qRT-PCR detection to reveal an inverse expression pattern between miR530 (up-regulation) and its target P2C37 (down-regulation) in middle-late developing SASK (Fig. 3b,c, and Additional File 9: Table S7), implying that miR530-directed P2C37 may be a major determinant of ABA response specific to early development of SASK. These results, together with growth reduction and oil accumulation of developing SASK observed mainly at middle-late stage (Fig. 1b,c), indicated that miR530-mediated ABA signaling pathway may repress SASK growth specifically at middle-late development, resulting in a high oil accumulation, as also noted in ABA-treated *C. vulgaris*^39^. It was reported that the activation of SnRK2s by ABA can phosphorylate ABA responsive element binding factor (ABF)^40^, one of which ABI4 can activate acyl-CoA: diacylglycerol acyltransferase 1 (DGAT1) for triacylglycerol (TAG) formation in Arabidopsis^41^. Recently, our mRNA transcriptomic analysis revealed that the highly expressed ABI4 and DGAT1 may play the important role in SASK oil accumulation at middle-late development^22^. In this work, both SnRK2A and SnRK2E were identified with the abundant transcript specifically in middle-late developing SASK (Additional File 9: Table S7), but only SnRK2A was predicted to be targeted by psi-miRn5 (Additional File 6: Table S4). Given the fact on the down-regulated transcript of psi-miRn5 and the similar up-regulated transcript profiles between ABI4/DGAT1 and SnRK2A/SnRK2E identified in middle-late developing SASK (Fig. 4 and Additional File 9: Table S7), we suggested that SnRK2E and psi-miRn5-targeted SnRK2A may involve in the regulation of ABI4 phosphorylation and DGAT1 activation for TAG assembly in developing SASK, which could be supported by our previous findings on a large accumulation of SASK oil at middle-late development^22^. In our opinion, the repression of P2C37 by miR530 may be critical for the active transcriptions of ABA-inducible genes (SnRK2E and psi-miRn5-targeted SnRK2A), resulting in a high expression of *DGAT1* at middle-late development of SASK, which may contribute to the rapid accumulation of SASK oils. Thus, miRNA-mediated ABA signaling pathway for SASK oil accumulation was mainly at middle-late stage.

It is interesting to note that over-expression of *ARF10*, a component of auxin signaling pathway, causes an up-regulation of ABA-inducible genes in Arabidopsis seeds, suggesting a cross-talk between auxin and ABA signaling pathways^42^. In the present investigation, the closely coordinated up-regulation of ARF1/9, and ABA-responsive SnRK2E and psi-miRn5-targeted SnRK2A was identified specifically at middle-late development of SASK (Additional File 9: Table S7). It is, therefore, considered that the up-regulated auxin-responsive ARF1/9 may participate in transcriptional regulation of some ABA-induced genes specifically at middle-late stage of SASK. Considering the rapid growth and a higher oil accumulation of SASK observed at early and middle-late development respectively (Fig. 1b,c), we postulated that a potential cross-talk between auxin and ABA signaling pathways may be prerequisite for development and oil accumulation of SASK. It was also noteworthy that ABA treatment could result in an increase of cytoplasmic Ca^2+^ in *Commelina communis* and *Vicia faba*^43^,^44^. The second messenger Ca^2+^ has been reported to be implicated in mediating responses to hormones signals, biotic/abiotic stress response and various developmental progresses in plants^45^. Here, it was found that contrary to the low-transcript of psi-miRn37 in late developing SASK, BAG6 (targeted by psi-miRn37), as one Ca^2+^-binding protein for the induction of programmed cell death in Arabidopsis^46^, displayed an up-regulated transcription (Figs 3b,c and 4). This investigation, integrated with the slow growth of SASK (Fig. 1b) and the strong ABA response specifically at late developing stage, reflected the potential relationship between psi-miRn37-mediated regulation and ABA signaling required for SASK maturity at late development. Taken together, the development and oil accumulation of SASK may be implicated in potential intercalations of different signaling pathways (ABA, auxin and Ca^2+^).

Recently, many TFs have shown to regulate the development, maturation and oil biosynthesis of plant seeds^47^,^48^. Importantly, some miRNA-targeted TFs have been identified to be involved in plant development or oil accumulation^13^,^26^,^27^,^49^. Indeed, by functional prediction and differential expression analysis, we identified many miRNAs (known and novel) with differential expression to target various groups of TFs in developing SASK (Additional File 6: Table S4 and Additional File 8: Table S6), implying an involvement of miRNA-targeted TFs in posttranscriptional regulation for developing SASK. It is well-known that miR156, as one conserved miRNA family, targets 11 *SPL* genes in Arabidopsis^27^. Here, we identified a negative correlation for miR156 family (down-regulation) and their targeted SPLs (up-regulation) at early development of SASK (Fig. 3a and Additional File 12: Fig. S4), indicating that miR156-mediated regulation of *SPL*s may implicate in developing SASK, as was reported in Arabidopsis^50^. Recently, co-expression network analysis has shown that *SPL* genes could activate other TF families (B3, bZIP, WRKY, MYB, bHLH, and MADs-box) in Arabidopsis^51^. Intriguingly, by our functional analysis of miRNAs, WIN1 (B3 family) targeted by psi-miRn24, WRKY7 by psi-miRn44, GAMYB (MYB family) by miR159 and psi-miRn31, and ATHB14 and 15 (HD-ZIP family) by miR166 were identified in developing SASK (Additional File 6: Table S4), emphasizing a complex regulatory network linking miR156-targeted *SPL* genes with other miRNA-targeted TFs in developing SASK. The fact that miR156-targeted SPL genes were characterized with a similar transcript pattern as miR171h-targeted SCL6 in developing SASK (Fig. 4), suggested that there exists one co-regulation mechanism of miR156-SPLs and miR171h-SCL6 in developing SASK, as previously noted in Arabidopsis^52^. Also, our findings on the up-regulated transcripts of miR156-targeted SPLs (Additional File 6: Table S4), and auxin-responsive factor (ARF2, 18 and 19) in developing SASK (Additional File 9: Table S7) indicated a potential interaction between miR156-targeted SPLs and auxin signaling in developing SASK, which was in accordance with previous studies of co-expression networks for SPL-correlated genes in Arabidopsis^51^. It has been reported that SPL genes may take part in regulation of carbohydrate transport, glucose metabolism and energy production in Arabidopsis^51^, and play the important roles in maize kernel development^53^ and tomato fruit ripeness^54^. Here, we observed that the abundant transcripts for ATVGT1, ATK, PGP4, ABCG18 and SULTR1;2 (as the downstream genes of SPL) specifically in early-middle developing SASK (Additional File 12: Fig. S4), displayed a coordinated temporal pattern with SASK growth (Fig. 1b) and gene expression of enzyme involved in substrate generation for FA biosynthesis^22^. Thus, it is undoubtedly clear that miR156-SPLs regulatory mode may form a complex co-expression network by the interplay of other miRNA-targeted TFs and auxin signaling pathway for SASK development and FA biosynthesis. Attentively, SPL9 activates directly miR172 transcription, and miR172 could repress downstream AP2, a negative regulator for oil deposition in Arabidopsis^55^,^56^,^57^. In this paper, 3 members (miR172b, miR172c and miR172d) of miR172 family exhibited a high expression in early developing SASK (Fig. 3a). The finding, together with an up-regulation of miR156-targeted *SPL* genes observed in developing SASK (Additional File 12: Fig. S4), prompt us to believe that the transcription of miR172 was positively regulated by miR156-targeted SPLs in developing SASK (Fig. 4). Importantly, *AP2*, as the target of miR172 (Additional File 8: Table S6), has been recently identified with up-regulation in early developing SASK^22^, suggesting that the translational inhibition of *AP2* gene may be by miR172 in developing SASK. All these results, combined with our previous investigations on oil accumulative pattern in developing SASK^22^, gave us some clear knowledge that the existence of regulatory pathway by the interactions between miR156-targeted SPLs and miR172-targeted AP2 may make an important contribution to control SASK development and oil accumulation, which was evidenced in Arabidopsis and maize^53^,^56^. Interestingly, miR482d, targeted the same gene *AP2* as miR172, was observed with a high expression in middle developing SASK (Fig. 4). Thus, both miR172 and miR482d may act as the negative regulator for *AP2* gene expression, which may play an important role in oil accumulation of developing SASK. Taken collectively, It is clear from both mRNA and miRNA transcriptome analysis that there exists a complex regulatory network of miRNAs and TFs in developing SASK, which may be responsible for the SASK development and oil accumulation.

In conclusion, our mRNA and miRNA transcriptome analysis revealed that the potential intercalations of different signaling pathways and a complex regulatory network of miRNAs and TFs in developing SASK, which may plays the critical roles in SASK development and oil accumulation. All our findings will make a great contribution to develop biodiesel for SASK in China.

## Methods

### Plant materials

Siberian apricot is widely distributed in China, so it has not been listed as an endangered or protected species and does not require approval. The different developmental stages of SASK were obtained from the same tree located at the Beijing Forestry University experimental station, Beijing, China. The developmental processes of SASK from flowering to seed maturity were observed from May to July 2015. Flowers with the same anthesis were marked, and then seeds were harvested at 10 DAF (immature stage), 20 DAF, 30 DAF, 40 DAF, 50 DAF, 60 DAF and 70 DAF (fully matured stage) respectively. After removing the sarcocarp, the seeds were immediately frozen in liquid nitrogen and stored at −80 °C until use. The SASK weight was weighed with electronic balance, and the SASK volume was investigated with the displacement method of drainage.

### Extraction and determination of SASK oil

The SASK from different developmental stages were crushed using a domestic grinder, producing a mean particle size of 0.8 mm, and the SASK oil was subsequently extracted with petroleum ether using a Soxhlet apparatus at 45–50 °C. After extraction for 6–8 h, the extracts were concentrated and dried and the solvent was then evaporated. The oil content was determined as the difference between the weights of the kernel samples before and after extraction. Extractions were performed in triplicate.

### Small RNA library construction and deep sequencing

According to the evaluative results of SASK oil variability for biodiesel properties, 3 crucial SASK samples (10, 30 and 60 DAF) were selected as materials for comparative sRNA sequence and analysis. The equal weight of three biological samples from every developmental stage was mixed, and then total RNA was extracted from the mixture using RNeasy Plant Mini Kits (Qiagen, Inc., Valencia, CA, USA) according to the manufacturer’s protocol. Extracted RNA was qualified and quantified using Nanodrop ND-1000 Spectrophotometer (Nanodrop Technologies, Wilmington, DE, USA), and all the samples showed a 260/280 nm ratio from 1.9 to 2.1. Two sRNA libraries (two independent biological replicates) from the same developmental stage of SASK were respectively used as samples to perform high-throughput sequencing by Illumina Hiseq2500. These data have been deposited in NCBI/SRA database under accession number of SRR2080279, SRR2097232 and SRR2097235.

### Bioinformatics analysis of sRNA data

After removing low quality reads and trimming adaptor sequences, the high-quality small RNA reads were obtained from the sRNA raw data. The unique reads were mapped to the peach genome by using the program Bowtie. Sequences that matched rRNA, scRNA, snoRNA, snRNA, or tRNA sequences in Rfam and NCBI GenBank databases (http://ftp.ncbi.nlm.nih.gov) were filtered out. The remaining sequences were searched against the miRBase database (http://www.mirbase.org) with up to one mismatch, to identify conserved miRNAs. The sequences that did not map to any miRNAs in miRBase were analyzed for predictions to identify novel miRNAs by the program miRDeep, and the RNA secondary structure of the predicted miRNAs was checked using RNAfold^25^. The potential targets of conserved and novel miRNAs were predicted using the psRNATarget program^58^. The previous *de novo* transcriptome assembly of developing SASK was used as the cDNA library for the target search^22^.

### Differential expression analysis of miRNAs in response to SASK development

The counts of identified miRNAs in 3 libraries were normalized as transcripts per million (TPM) according to the formula: Normalised expression = actual miRNA count/total count of clean reads × 1,000,000. The normalized reads were used to calculate *p*-value and Fold-change in expression abundance. The differential expression of miRNAs between 2 libraries was calculated as: Fold-change = log_2_ (value 1/value 2). The *p*-value was obtained according to previously reported methods^59^. The miRNAs with absolute values of log_2_ (ratio) > 2, along with *p*-value < 0.05, were considered with significantly differential expression.

### Differential expression analysis of target genes

Based on the previous 5 cDNA libraries (SRR1564517, SRR1568273, SRR1568275, SRR1568789 and SRR1568805)^22^, the expression levels of target genes was calculated using RPKM (Reads per kilobase transcriptome per million mapped reads). The levels of unigenes expression in the different samples were compared using the DESeq method^59^.

### qRT-PCR analysis of miRNAs and their targets

The RNA was extracted as the description for sRNA library preparation. The fist-strand cDNA synthesis of miRNAs and their targets was performed by respectively using miRcute miRNA first-strand cDNA synthesis kit (TIANGEN) and reverse transcription System (Promega). The qRT-PCR of miRNAs and their targets was performed by respectively using the miRcute miRNA qPCR deection kit (TIANGEN, Beijing, China) and the SYBR Premix *Ex Taq* Kit (TaKaRa) according to the manufacturer’s protocol in an ABI 7500 fast system. The 5.8S rRNA and ubiquitin-conjugating enzyme (UBC) gene were used as internal controls for qRT-PCR analysis of miRNAs and their targets, respectively. Three technical repetitions were performed for qRT-PCR. The primer sequences are shown in Additional File 13: Table S9.

## Additional Information

**How to cite this article**: Niu, J. *et al.* Integrated mRNA and miRNA transcriptome reveal a cross-talk between developing response and hormone signaling for the seed kernels of Siberian apricot. *Sci. Rep.* **6**, 35675; doi: 10.1038/srep35675 (2016).

## Supplementary Material

Supplementary Information

Supplementary Table S1

Supplementary Table S2

Supplementary Table S3

Supplementary Table S4

Supplementary Table S5

Supplementary Table S6

Supplementary Table S7

## Figures and Tables

**Table 1:** Summary of cleaning data from three sRNA libraries.

Sample (DAF)	Total read	Reads trimmed adaptor	Reads trimmed N	Reads trimmed quality dynamics	Reads trimmed Quality Percent	Reads trimmed PolyA/T	Reads Trimmed length	Clean data	Unique reads
10	8,173,971	8,055,861	19,458	25	394	2267	717,028	7,336,170	1,905,120
30	8,007,157	7,861,276	18,132	25	271	1169	575,730	7,284,101	2,116,046
60	9,214,139	8,986,857	14,243	25	940	1261	1,547,581	7,437,072	1,599,564

**Table 2:** Novel or candidate miRNAs identified from SASK sRNA libraries.

miRNA	miRNA sequence (5′-3′)	Count (DAF)			miRNA* sequence (5′-3′)	Count (DAF)			Precursor location
		10	30	60		10	30	60	
miRn1	ccugcgucgcuucgauucgu	5048	29428	40015	auacgcgacgggguauugua	185	374	110	scaffold_4_contig_311:84303..84385:−
miRn2	agauuuccuggaauuguauaacuc	0	7	20	auuauagacuuccauacuuuugua	0	0	2	scaffold_8_contig_172:135475..135567:−
miRn3	aagagaguguaagugaacaaaaga	9	22	11	ccuuuuguccacuuacacucc	4	1	3	scaffold_7_contig_204:666903..666996:−
miRn4	agagaaaugacuccuaguggcucc	86	75	16	agacuucuaagagcuccuaaaucu	1	1	1	scaffold_8_contig_45:17360..17414:+
miRn5	uugagugcagcguugaugaau	90	4	10	uuucaucagcgcugcacccaa	24	3	5	scaffold_4_contig_15:318297..318355:+
miRn6	auugaucgaggauagcaaaacagu	20	25	18	uuugcuaucuccaaccaauaacgc	0	0	1	scaffold_8_contig_59:232853..232940:+
miRn7	cuaccgauuccacccauuccga	8086	11845	15623	ggauuggguggguuugguaaga	2	3	4	scaffold_1_contig_329:320383..320460:+
miRn8	aaaacggcgucguuuuggaccagc	37	31	8	uggccaaaacggcgucauuuugg	0	1	0	scaffold_3_contig_29:28021..28105:+
miRn9	agaaggggacuguauauauauaua	491	354	28	auauauauaugcgggccuaauggg	1	0	0	scaffold_1_contig_372:35009..35058:+
miRn10	ugacgacgagagagagcacgc	19	285	5509	ugcucucucuuguugucauaca	0	6	3	scaffold_8_contig_182:345266..345349:+
miRn11	uuuagggaguccauuguagaugcu	7	55	180	aguugcauuuuuacgauuccuccc	1	6	0	scaffold_6_contig_83:51950..52021:+
miRn12	agauaaacuuuugagcuuggcaug	135	113	13	auguggcaugggagcaaaaucaac	3	1	0	scaffold_7_contig_100:157993..158047:−
miRn13	uaagguugagccggaaaucgga	23	0	5	cgacucccgcucaaucucaug	7	0	0	scaffold_6_contig_93:110629..110716:+
miRn14	auuggucggggauagcaaaauagu	44	51	5	acuauuuugcuauccccggucaau	0	0	1	scaffold_4_contig_138:6339..6431:+
miRn15	uugcaugggccuggcgcacccca	113	51	106	ucucaagcgucgcccaagcguu	7	6	4	scaffold_1_contig_96:40220..40271:+
miRn16	ugccaagaaagaguugcccua	62	24	8	uagggcuccucuuucuuggca	40	25	17	scaffold_3_contig_12:291955..292046:−
miRn17	uuuccgaaaccucccauuc	0	42	201	gggugagagguugccggaaaga	2	24	0	scaffold_1_contig_329:321818..321895:+
miRn18	ucuuuccuacuccacccauuc	113	490	3056	agugggagagugggaaaagaaug	29	25	5	scaffold_1_contig_329:327349..327425:+
miRn19	aaggaccaacuugauguaccaaga	11	46	24	ucuugguacaucaaguugguc	0	1	0	scaffold_2_contig_32:89354..89424:−
miRn20	ugauuggucgaggauagcaaa	94	296	27	cgcuauuuucggccaaucaca	1	0	0	scaffold_3_contig_215:374526..374606:−
miRn21	augacccaaaagugcuucuaaacu	0	0	1	aagcgcuuuugguugucagaaag	33	14	18	scaffold_4_contig_37:135453..135520:−
miRn22	cauggaucaggccucagagaa	6	1	10	cuccauguaaggcuagccgaa	39	11	27	scaffold_4_contig_122:94489..94536:+
miRn23	auuauggacucauggguauggacu	7	75	18	uucacacccuuggucaccuugga	0	0	0	scaffold_10_contig_3:378164..378229:−
miRn24	auccccgaccaauaacacaaugac	56	20	14	cauugccuuaguggccggggauag	0	0	0	scaffold_6_contig_239:229691..229763:+
miRn25	auuuguggaaccaugguuaugccc	36	31	9	gcauaaccauaguuaacucuuug	0	0	0	scaffold_3_contig_54:24274..24327:−
miRn26	aguggauauguuauuauguggccu	22	0	0	uuguaaauguuauucuguggccu	0	0	0	scaffold_6_contig_184:109044..109110:+
miRn27	ugauucuuggugacgugaugu	426	669	766	aucaugucaccaggaaccaag	0	0	0	scaffold_7_contig_33:88214..88295:−
miRn28	accacgugacuguauuccaguacc	23	38	20	uauaggaguaugaugaggugguau	0	0	0	scaffold_6_contig_55:15340..15426:−
miRn29	uucacacucgucaaauuccaacag	0	23	64	aauggauauaauaaugagugaugauagg	0	0	0	scaffold_6_contig_256:242480..242568:+
miRn30	ugagucaggaaugaguagcc	596	500	26	ggcuugcucauuccugacu	0	0	0	scaffold_4_contig_145:37389..37467:−
miRn31	uuuggacugaagggagcuccc	686	92	124	gugcuccuugcagcccaaaac	0	0	0	scaffold_7_contig_229:39979..40065:+
miRn32	auuagucgggaauaacaaaacaau	14	57	23	auuauuuugcuauuuucggccaau	0	0	0	scaffold_1_contig_314:198611..198703:−
miRn33	uugccaaccccgcccauuccaaa	954	1911	4823	uggaaugagcguguuggaaaag	0	0	0	scaffold_1_contig_329:324015..324086:+
miRn34	agagagaugauacacaaguugguc	38	52	31	ccaaauuguauaucauuuuucuaa	0	0	0	scaffold_6_contig_39:200857..200946:+
miRn35	aauacucgugcgucugugaaaagu	44	46	9	cgcgcgcagacugcucgacuaaggg	0	0	0	scaffold_5_contig_39:123346..123399:−
miRn36	auuuaaaugggcugggcucgugcc	21	37	2	ccagcucauccuauuuggcacc	0	0	0	scaffold_4_contig_304:85169..85223:−
miRn37	ucuggugaaucucuaauucgau	20	0	9	cgauuagugaugcaccagaaa	0	0	0	scaffold_5_contig_68:76608..76681:−
miRn38	ugacaacgagagagagcacg	1	9	214	ugcucucucuuguugucaug	0	0	0	scaffold_8_contig_182:348995..349076:+
miRn39	aacauuauuuuguuauuuccggcc	40	38	8	ccggcauagcaaaauaguguuau	0	0	0	scaffold_4_contig_178:88999..89090:−
miRn40	auuccagcaccuguucauggcccu	24	5	3	agcuuaugaauuugauuugugg	0	0	0	scaffold_4_contig_240:6099..6167:−
miRn41	ccaggaaucaaguugggaccu	211	106	209	gccccaacuugauuucuauug	0	0	0	scaffold_8_contig_92:147159..147245:−
miRn42	augagauauuggaggaaacuaaac	0	33	11	uuaguccccuccaauucucacau	0	0	0	scaffold_3_contig_223:476238..476327:−
miRn43	uuauacaaugaaaucacggcc	4495	5507	12402	ccguguuucuuuguauaaa	0	0	0	scaffold_1_contig_27:6023..6110:+
miRn44	uuauuggucggggauagcaaa	107	45	172	uugcuauccucagccaauaaug	0	0	0	scaffold_4_contig_214:333063..333144:−
