# Supplementary material for: Integrated mRNA and miRNA transcriptome reveal a cross-talk between developing response and hormone signaling for the seed kernels of Siberian apricot
Source: Sci Rep. 2016 Oct 20;6:35675. doi: 10.1038/srep35675 (PMC5071837; doi:10.1038/srep35675)
Supplement: Supplementary Information [file srep35675-s1.doc]

**Integrated mRNA and miRNA transcriptome reveal a cross-talk between developing response and hormone signaling for the seed kernels of Siberian apricot**

**Jun Niu^1a^,** **Jia Wang^1a^, Jiyong An^1^, Lili Liu^1^, Zixin Lin^1^, Rui Wang^1^,** **Libing Wang^2^, Chao Ma^1^, Lingling Shi^1^, Shanzhi Lin^1b^**

^1^College of Biological Sciences and Biotechnology, National Engineering Laboratory for Tree Breeding, Key Laboratory of Genetics and Breeding in Forest Trees and Ornamental Plants, Ministry of Education, Beijing Forestry University, Beijing 10083, China

^2^Research Institute of Forestry, Chinese Academy of Forestry, Beijing 10091, China

^a^These authors contributed equally to this work

^b^Corresponding author: Tel/Fax +86-10-62336114; [szlin@bjfu.edu.cn](mailto:szlin@bjfu.edu.cn)

**Additional file 1: Fig. S1. The quality evaluation of sRNA sequencing data.** The green means "PASS", yellow means "WARN" and red means "FAIL".

**
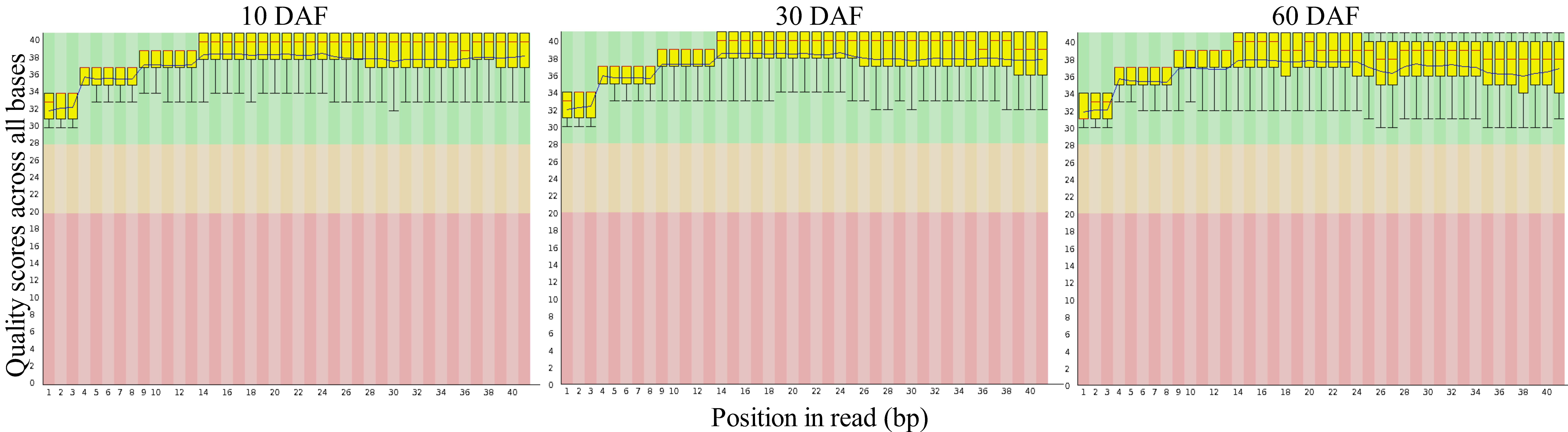
**

**Additional file 2: Table S1. Distribution of sRNAs among different categories in SASK.**

| **Total sRNA class** | **10DAF** | **%** | **40 DAF** | **%** | **60 DAF** | **%** | **Uniq sRNA class** | **10 DAF** | **%** | **40 DAF** | **%** | **60 DAF** | **%** |
| --- | --- | --- | --- | --- | --- | --- | --- | --- | --- | --- | --- | --- | --- |
| **rRNA** | 583196 | 7.95% | 997918 | 13.70% | 1649747 | 22.18% | **rRNA** | 50220 | 2.64% | 57004 | 2.69% | 63682 | 3.98% |
| **snoRNA** | 57554 | 0.78% | 59704 | 0.82% | 82261 | 1.11% | **snoRNA** | 8235 | 0.43% | 7971 | 0.38% | 12182 | 0.76% |
| **repeat** | 9888 | 0.13% | 18934 | 0.26% | 25128 | 0.34% | **repeat** | 2813 | 0.15% | 3801 | 0.18% | 4064 | 0.25% |
| **miRNA** | 974329 | 13.28% | 478734 | 6.57% | 564848 | 7.60% | **miRNA** | 2105 | 0.11% | 2496 | 0.12% | 2942 | 0.18% |
| **tRNA** | 128677 | 1.75% | 249191 | 3.42% | 468066 | 6.29% | **tRNA** | 10110 | 0.53% | 11503 | 0.54% | 14420 | 0.90% |
| **snRNA** | 16116 | 0.22% | 17814 | 0.24% | 20962 | 0.28% | **snRNA** | 4598 | 0.24% | 4922 | 0.23% | 5631 | 0.35% |
| **other** | 5566410 | 75.88% | 5461806 | 74.98% | 4626060 | 62.20% | **other** | 1827039 | 95.90% | 2028349 | 95.86% | 1496643 | 93.57% |
| **Total** | 7336170 | 100% | 7284101 | 100% | 7437072 | 100% | **Total** | 1905120 | 100% | 2116046 | 100% | 1599564 | 100% |

**Additional file 3: Table S2. List of know miRNAs identified in developing SASK and their normalized reads in 3 sRNA libraries.**

**Additional file 4: Table S3. All SASK-specific pre-miRNA sequences, secondary structures and minimal folding energies.**

**Additional file 5: Fig. S2. Predicted secondary structure of some SASK-specific pre-miRNA sequences. The red indicate the mature miRNA sequence.**


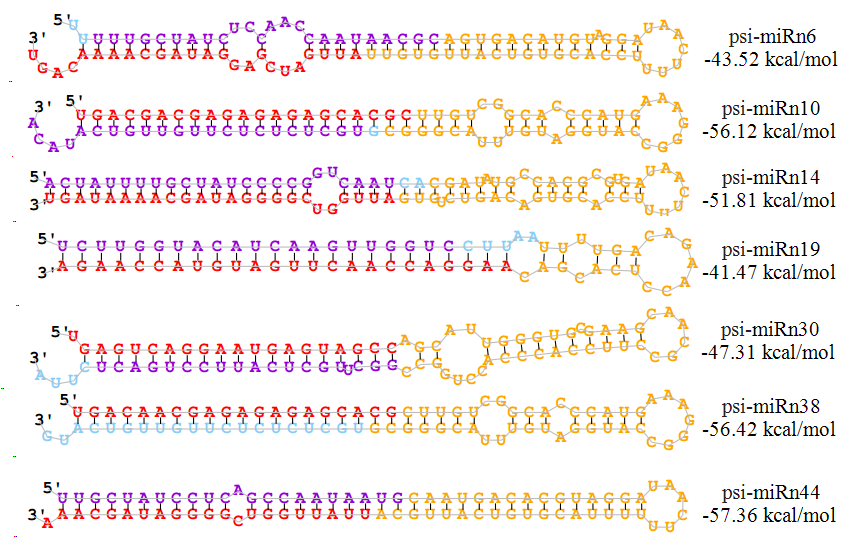


**Additional file 6: Table S4. Predicted targets for known and novel miRNAs, and the functional annotations and expression levels for targets.**

**Additional file 7: Table S5. The differential expression miRNAs in 30/10 DAF, 60/10 DAF and 60/30 DAF.**

**Additional file 8: Table S6. The summary of differential-expression miRNAs and their targets.**

**Additional file 9: Table S7. List of all genes and differential-expression genes involved in plant hormone signal transduction.**

**Additional file 10: Fig. S3. The KEGG map of plant hormone signal transduction.**


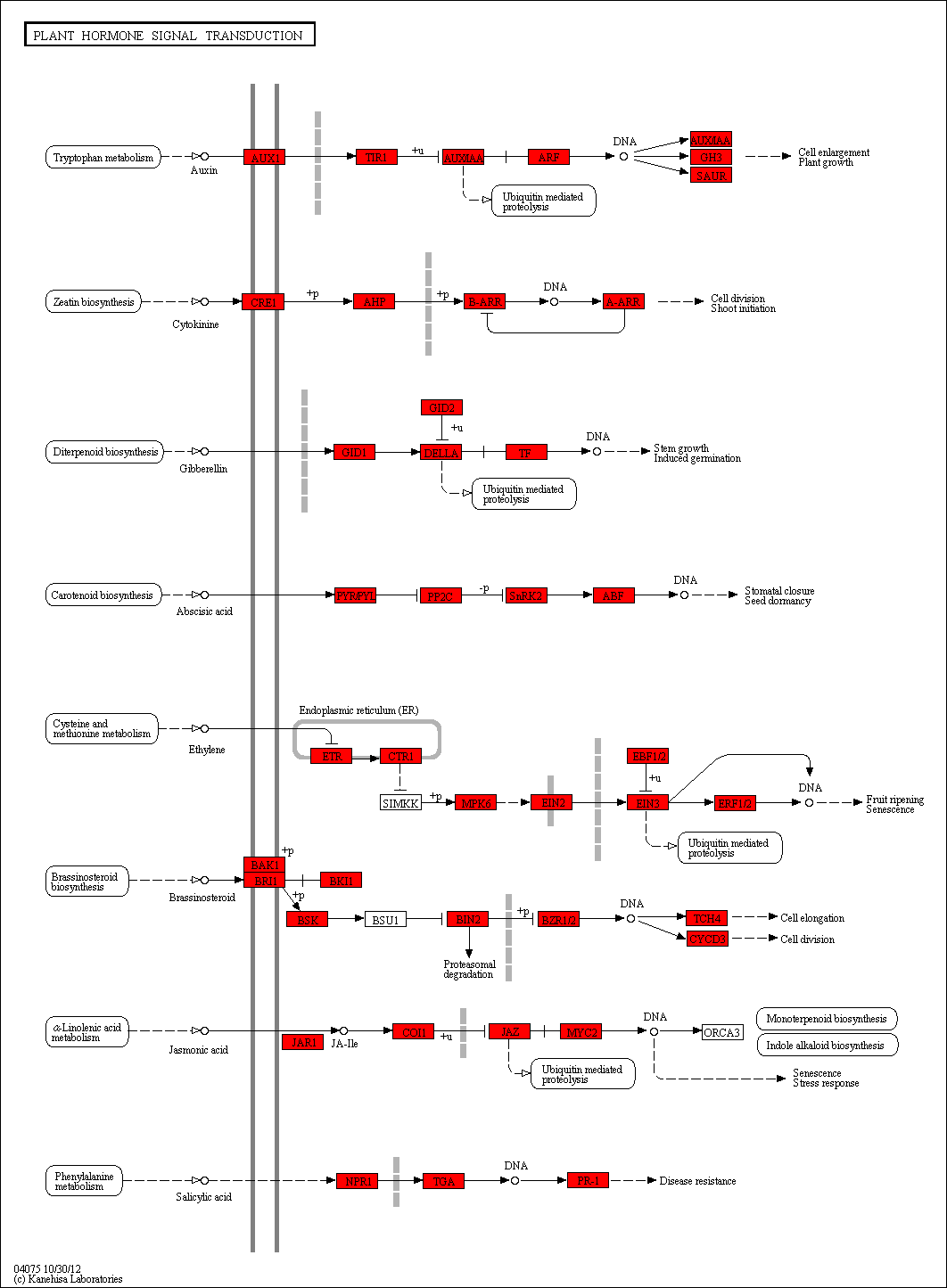


**Additional file 11: Table S8. The miRNAs involved in plant hormone signaling pathways.**

**Additional file 12: Fig. S4. The expression levels of SPLs and their downstream genes.**

**
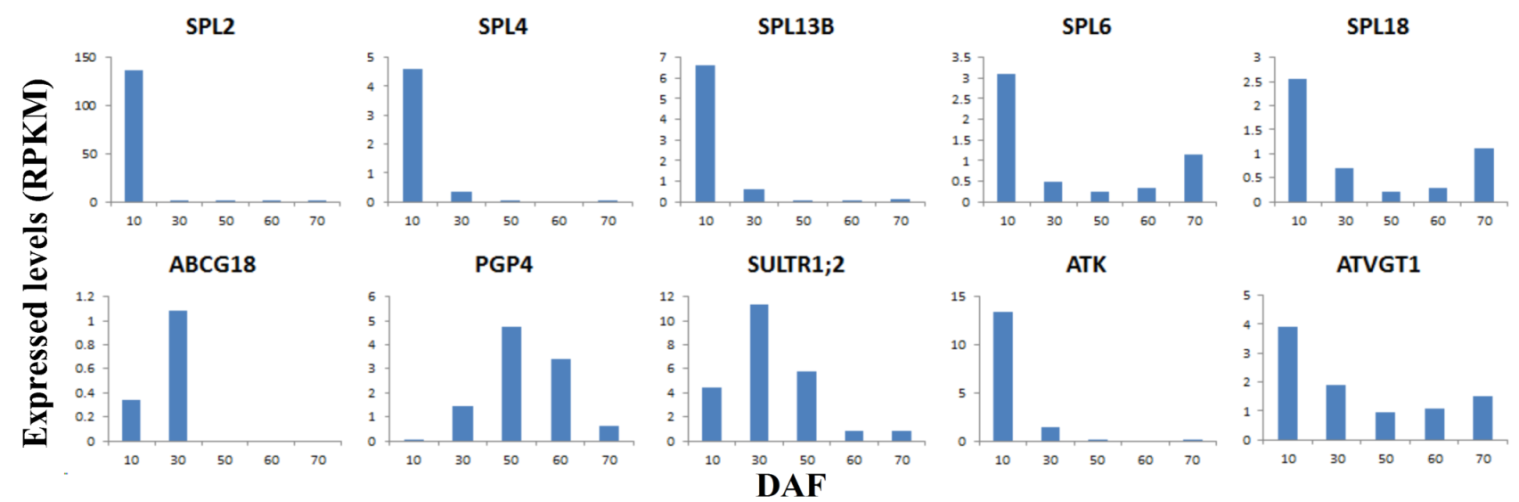
**

**Additional file 13: Table S9** List of primers used in this study for qRT-PCR of miRNAs.

| MiRNAs | Seqtence (5’-3’) | Targets | Sense Primer (5’-3’) | AntiSense Primer (5’-3’) |
| --- | --- | --- | --- | --- |
| miR156a | TGACAGAAGAGAGTGAGCAC | comp47180_c0 | GTCTATGGCAAGGCCATCTT | TGAACACCGCAGACCTTATG |
| miR160h | TGCCTGGCTCCCTGTATGC | comp70106_c0 | CTCTCGGTTCTTGGCTCTTATG | CCTGTCACATCCCGGTAAAG |
| miR164a | TGGAGAAGCAGGGCACGTG | comp65068_c0 | CCGTTCCCAGGTTCAGTTT | TGGGACACACTGACCATTTC |
| miR171h | TTGAGCCGCGTCAATATCTC | comp55922_c1 | GCCCTCCAGTCCTACATAAAC | CCCTAGAACAGTGCTCTCAATC |
| miR393h | TTCCAAAGGGATCGCATTGA | comp72820_c0 | ATGGCAGCGAGCTGATAAA | CTCCTAGTCCTTTGTCTCCAATAC |
| miR395a | CTGAAGTGTTTGGGGGAACT | comp54000_c0 | CGGAGGGTTGTGGGATTTAT | AACATGTCTTCCAGGGCTTTA |
| miR530 | CTGCATTTGCACCTGCACC | comp37670_c0 | GATGTCGGTTTGTGGGAGAA | CCATAGAAGTGGGCTTCATTAGA |
| miR6281 | GTTAGAGATAGAGAGAGTGAG | comp62250_c0 | CTGCTCTCTCGCATCTTTGT | CGTAAGCTCTCTAGGCAAGTTC |
| psi-miRn5 | TTGAGTGCAGCGTTGATGAAT | comp78360_c0 | TATCCAGGAAGCCAACCATTC | CGTCTCTCTGGGCTTCTTTATC |
| psi-miRn37 | TCTGGTGAATCTCTAATTCGAT | 5.8s rRNA | CTCGGCAACGGATATCTCG | CTAATGGCTTGGGGCG |
|  |  | UBC | GAGACCAGCAATAACCGTGAA | TCTTGTACTCCGTGGCATCCT |
